# Addressing Vaccine Hesitancy: Validating the PACV Survey for Croatian Parents

**DOI:** 10.3390/idr17010003

**Published:** 2025-01-08

**Authors:** Ana Ćurković, Antonela Matana

**Affiliations:** University Department of Health Studies, University of Split, Ruđera Boškovića 35, 21000 Split, Croatia; acurkovic@ozs.unist.hr

**Keywords:** vaccine hesitancy, parent attitudes about childhood vaccines (PACV), Croatia, validation

## Abstract

**Background/Objectives:** Vaccine hesitancy, recognized by the WHO as a significant global health threat, undermines vaccination efforts. This study aimed to adapt and validate the Parent Attitudes about Childhood Vaccines (PACV) Survey for Croatian parents to understand vaccine hesitancy better. **Methods:** A cross-sectional study with 1814 Croatian parents was conducted using the PACV survey, translated using a double-back translation method. The psychometrics of the questionnaire were examined, including content validity, dimensionality, construct validity, discriminant validity, and reliability. Confirmatory factor analysis (CFA) assessed the original model’s validity, but the original model fit poorly, prompting exploratory factor analysis (EFA) to identify latent factors. Reliability was measured using Cronbach’s alpha and McDonald’s omega. **Results:** EFA identified a five-factor model with factors labeled “Safety”, “Schedule”, “Attitudes”, “Behavior”, and “Trust”. Subsequent CFA confirmed this model with acceptable to good fit indices. Discriminant validity was also confirmed. Reliability analyses indicated high internal consistency, with total Cronbach’s alpha at 0.926 and McDonald’s omega at 0.931. **Conclusions:** The adapted PACV is a valid and reliable tool for identifying vaccine hesitancy among Croatian parents. This study revealed high vaccine hesitancy among Croatian parents, emphasizing the need for targeted interventions.

## 1. Introduction

Vaccine hesitancy can reduce vaccination coverage, diminishing the collective immunity of the population and leading to outbreaks of vaccine-preventable diseases [1,2]. Outbreaks of measles, mumps, and pertussis have been observed in the US [3], while measles outbreaks have occurred in Europe [4]. These are the reasons why the World Health Organization has included vaccine hesitancy among the top 10 global health threats [5]. Vaccine-hesitant parents exhibit a range of behaviors, such as refusing some vaccines, delaying their children’s vaccinations, or accepting vaccinations with uncertainty [6]. In the general population, there are a greater number of parents who are hesitant about vaccinating their children, as opposed to those who refuse vaccines [7]. Therefore, it is crucial to understand vaccine hesitancy to address the decrease in childhood vaccination rates [8,9]. Delaying or refusing vaccination poses a risk not only to unvaccinated children but also to the entire community if childhood vaccination rates drop [10]. In Croatia, vaccination is mandatory for all children without contraindications since 1948, when mandatory vaccination against tuberculosis (BCG) and diphtheria was introduced [11]. Over the years, new vaccines against various infectious diseases have been added to the mandatory vaccination program, which today includes vaccination against 11 infectious diseases (tuberculosis, diphtheria, tetanus, pertussis, polio, measles, mumps, rubella, hepatitis B, diseases caused by Haemophilus influenzae type B, and pneumococcal disease) [12]. Based on the guidelines of the Croatian Institute of Public Health [11,13], Croatia’s state-sponsored program ensures that all children receive these vaccines up to the age of 18, with all expenses covered by the government [12]. If a parent refuses to fulfill their obligation to vaccinate their child, they may face a monetary fine under the Law on the Protection of the Population from Infectious Diseases [14]. However, according to recent data from the Croatian Institute of Public Health, Croatia has not achieved the recommended 95% vaccination rate for achieving community immunity for any vaccine except for the BCG vaccine administered to newborns before they are discharged from the hospital after birth, which has a coverage rate of 97.1% [15]. According to a population study in 2016, 11% of Croatian parents refused all mandatory vaccines for their children, while 20% agreed to have their children vaccinated with some mandatory vaccines [16].

In recent years, some studies have recorded changes in parental attitudes toward vaccination and an increase in childhood vaccine hesitancy [17,18]. A Turkish study showed that vaccine hesitancy grew as the COVID-19 pandemic progressed [19]. Some research has indicated that, since the start of the COVID-19 pandemic, parents have become more concerned about vaccine safety [17,20,21] and potential side effects [20]. In certain studies, more negative attitudes regarding vaccine safety and efficacy have been observed after the onset of the pandemic [17,21], along with decreased overall trust in vaccines [22]. More negative attitudes have been recorded in areas with lower COVID-19 vaccination coverage [21].

Accordingly, during the pandemic, a decline in routine childhood vaccinations was recorded worldwide [23,24,25,26,27], leading to decreased immunization coverage against infectious diseases. The largest drop was observed in middle-income countries [26]. In Croatia, during the COVID-19 pandemic, a decrease in both preventive check-ups and childhood vaccinations was also recorded [28]. According to the Croatian Health Statistics Yearbook for 2023 [29], vaccination coverage for certain vaccines decreased by 0.4% to 8.9% compared to 2019 [30], indicating an overall decline in immunization rates in the post-pandemic period. This downward trend in vaccination coverage may be linked to increased distrust of vaccines during the pandemic [17,21] and disruptions in routine immunization programs caused by COVID-19 [28].

Since vaccine hesitancy has emerged as one of the primary factors contributing to lower vaccination rates [17,18], a comprehensive understanding of vaccine hesitancy is crucial for the development of strategies aimed at increasing childhood vaccination rates [19]. The recorded decline in childhood immunization in Croatia [29] poses a significant risk to public health, making it important to address the issue of vaccine hesitancy.

The Parent Attitudes about Childhood Vaccines (PACV) Survey [31] is designed to detect vaccine-hesitant parents. The original was developed in American English in the United States by Opel et al. [31,32]. The survey is designed for self-administration, with a readability level equivalent to the 6th grade, and can be completed in less than 5 min. Originally, it consisted of 18 items categorized under four domains: (1) “Immunization behavior”, (2) “Beliefs about vaccine safety and efficacy”, (3) “Attitudes about vaccine mandates and exemptions”, and (4) “Trust” [31]. When the construct validity and reliability of the questionnaire were assessed, three items from the PACV were deleted [32]. The final version of the PACV questionnaire consisted of 15 items grouped into three domains: (1) “Safety and efficacy”, (2) “General attitudes” and (3) “Behavior”. The internal consistency of the original survey was acceptable [32]. Research also demonstrates that the parental PACV score correlates with the number of days children are under-immunized, confirming its construct validity [32].

The original PACV questionnaire has been translated and validated into several languages worldwide and across Europe [33,34,35,36,37]. In most validation studies [33,34,35,36,37], a three-factor structure was identified through factor analysis, with variations in item loadings within sub-domains. Generally, the identified factors were General Attitudes, Safety and Efficacy, and Behavior. For the Vietnam version, the factors were General Attitudes, Safety and Efficacy, and Children and Vaccination [35]. For the Malay version, the factors were General Attitudes, Safety and Efficacy, and Schedule and Immunity [36].

We consider the translation and validation of the PACV questionnaire for the population of Croatian parents an important step necessary for understanding vaccine hesitancy in Croatia. To our knowledge, the survey has not yet been applied or validated in the Croatian population. Therefore, the objective of this study was to adapt and translate the original PACV questionnaire from English to Croatian language and to examine its psychometrics, including content validity, dimensionality, construct validity, discriminant validity, and reliability. Sensitivity to change was not assessed, as it was not considered relevant for this study, given its focus on attitudes rather than clinical outcomes [38]. By validating the questionnaire specifically for the Croatian population, we ensure that it can effectively assess the attitudes, beliefs, and concerns of Croatian parents about childhood vaccination, providing valuable insights into the specific factors contributing to vaccine hesitancy in this context.

## 2. Materials and Methods

### 2.1. Study Design

This cross-sectional study was conducted in 2023, from March to November. A questionnaire was administered to a convenient sample of 1814 parents who accessed the questionnaire through a Google Forms link distributed with an invitation to participate in the study on social media. The convenient sample refers to participants who were easily accessible and willing to take part in the study, with the sample being drawn from those who voluntarily accessed the survey link shared in various Croatian parent-focused social media groups. The invitation and explanation of the research purpose were written in Croatian and shared across these groups. To ensure completeness, the survey was designed to require responses to all questions before submission, eliminating the possibility of incomplete entries. Adults were included if they had at least one child, whereas adults without children and minors were excluded. The upper age limit of parents as respondents and the children to whom the questionnaire referred was not defined because Croatia’s mandatory vaccination program covers children from birth until the completion of primary school and includes checking vaccination status and administering missed vaccines at the ages of 19 and 24 [12]. All participants were informed about the purpose and objectives of the research. Participation was voluntary and anonymous, with the option to withdraw at any time. Pressing the submit button was considered informal consent to participation in the research. The research was approved by the Ethics Committee of the University Department of Health Studies (class: 029-03/23-08/01, reg.no.: 2181-228-103/1-11, approved on 22 March 2023 and class: 029-03/23-08/01, reg.no.: 2181-228103/1-11, approved on 27 September 2023).

### 2.2. PACV Questionnaire

The questionnaire consists of 15 questions with three different response formats (3 questions with dichotomous yes/no responses, 10 questions with a 5-point Likert scale, and 2 questions with an 11-point scale). To determine vaccine hesitancy, the total PACV score was calculated using the original method developed by the authors of the questionnaire, Opel et al. [31,32]. The responses to the 15 questions were grouped into three categories: non-hesitant (assigned 0 points), unsure (assigned 1 point), and hesitant (assigned 2 points). These categories resulted in the PACV score, which can range from 0 to 30. Following the original method provided by the authors, the PACV score was linearly transformed into a range of 0 to 100. A score <50 indicated non-hesitancy, while a score ≥50 indicated hesitancy. Additionally, the questionnaire included some socio-demographic and general questions, such as gender, age, marital status, level of education, and number of children.

### 2.3. Questionnaire Translation, Adaptation, and Content Validation

The PACV questionnaire was translated from English to Croatian using a double back-translation method [39] to ensure content validity. An expert in the field and an English language professor independently translated the questionnaire into Croatian. After that, another expert in the field and another English language professor conducted two back-translations into English. To ensure the meaning of the questionnaire was preserved, the two English versions resulting from the back-translation were compared to the original English version by the research team. Any discrepancies or differences in wording were carefully reviewed, discussed, and resolved through consensus to ensure the translated version reflected the original content accurately. Finally, the two Croatian versions were integrated into one to create the final version of the questionnaire through a focus group discussion involving all participants in the translation process, during which consensus was reached on the best wording and structure. We obtained the author’s permission to use and translate the questionnaire.

### 2.4. Dimensionality and Construct Validation of the Questionnaire

We performed factor analyses to test the dimensionality and construct validity of the questionnaire. Dimensionality refers to the identification and organization of underlying factors or components that group related items within a questionnaire, while construct validity is defined as the extent to which the questionnaire accurately measures the theoretical concept it is intended to assess [40]. First, we tested the original three-dimensional structure developed by Opel et al. [32] by using confirmatory factor analysis (CFA), a statistical method used to evaluate whether a set of observed variables aligns with a predefined factor structure. The model fit adequacy was assessed by standard fit indices: χ2-statistics, the Root Mean Square Error of Approximation (RMSEA), and the Standardized Root Mean Square Residual (SRMR) with values <0.10 considered acceptable, and values ≤0.05 as good fit. Further, the Comparative Fit Index (CFI) and the Tucker–Lewis Index (TLI) were reported with an acceptable fit at 0.90 and a good fit at 0.95 [41]. Since χ2-statistics is sensitive to sample size, using this measure along with other fit measures is recommended. Therefore, we examined the ratio χ2/df. Recommendations for χ2/df range from as high as 5.0 [42] to as low as 2.0 [43]. Utilizing CFA, the high loading of an item signifies that the item strongly reflects the corresponding factor, while lower loadings suggest weaker relationships between the factor and the respective item. Factor loadings < 0.32 are considered poor, whereas those ≥ 0.45 are categorized as fair, ≥ 0.55 as good, ≥0.63 are regarded as very good, and those >0.71 are considered excellent.

Since the original three-dimensional structure had a poor fit to our data, we additionally performed an exploratory factor analysis (EFA) on our data set. We aimed to investigate the factor structure of the questionnaire and reveal the latent factors underlying the parent attitudes about childhood vaccines in Croatia without predefined hypotheses. Furthermore, we performed CFA on our dataset to assess whether the hypothesis proposed in the exploratory step is plausible in the population.

We have divided the total dataset into training (70%, *n* = 1304) and test (30%, *n* = 560) sets by a random selection using R software (version 4.1.0). The EFA was performed on the training set. Kaiser–Meyer–Olkin (KMO) measure of sampling adequacy and Bartlett’s test of sphericity were used to assess the suitability of the sample for factor analysis. Multivariate normality was tested with Mardia’s Test. We also tested factor correlations. Since data were not normally distributed and factors were correlated (correlation among factors ranged from 0.569 to 0.752), the minimum residual extraction method was used in combination with a cluster rotation. The number of factors to be extracted was estimated using parallel analysis and the examination of the scree plot. In this study, the model fit adequacy was assessed by χ2–statistics, χ2/df, RMSEA, SRMR, CFI, and TLI.

Subsequently, CFA was conducted on the test set, adhering to the previously described procedure, to validate the proposed factor structure identified through EFA.

### 2.5. Discriminant Validation of the Questionnaire

Discriminant validity, a measure that refers to the degree to which a construct is truly distinct from other constructs, was assessed using the heterotrait-monotrait ratio (HTMT). An HTMT value below 0.9 indicated good discriminant validity [44].

### 2.6. Reliability of the Questionnaire

Finally, to test scale reliability, we calculated the reliability coefficients of Cronbach’s alpha and McDonald’s omega to assess the internal consistency of the items. Internal consistency refers to the extent to which items within the questionnaire are correlated and measure the same underlying construct. Usually, both coefficients are considered adequate when above 0.70. However, in exploratory research, Cronbach’s alpha ≥ 0.6 is considered acceptable [45].

### 2.7. Descriptive Statistics

We used descriptive statistics to summarize the socio-demographic characteristics of the participants, responses to PACV items, and PACV score. Categorical variables are presented with frequencies (percentages). Due to the normal distribution of the data, continuous variables are presented with the mean and standard deviation (SD).

Descriptive statistics were performed with the IBM SPSS version, while other analyses were conducted using JASP (Version 0.18.3) [Computer Software] (Available online: https://jasp-stats.org/faq/, accessed on 28 October 2024).

### 2.8. Statistical Power Analysis

We conducted a post hoc power analysis utilizing the R package semPower (available online: https://cran.r-project.org/web/packages/semPower/readme/README.html, accessed on 28 October 2024) [46]. For the CFA, we calculated the study’s power based on *N* = 1816, df = 87, significance level = 0.05, and RMSEA = 0.05, yielding a power value exceeding 0.9999. Similarly, for the EFA, the power was greater than 0.9999, based on *N* = 1304, df = 40, significance level = 0.05, and RMSEA = 0.05.

## 3. Results

A total of 1868 parents responded to the invitation and completed the survey. Of these, 54 respondents were excluded because they did not have children and therefore did not meet the inclusion criteria. Therefore, the final number of respondents was 1814. The majority of participants were mothers (95.9%), married, or in a common-law relationship (94.9%). The average age was 35 years, and slightly more than half of the respondents had higher education (57. 1%). Most respondents had two children (44.2%) or one child (35.3%) (Table 1).

Nearly half of the respondents (48.4%) had a total PACV score of 50 or higher, classifying them as vaccine-hesitant parents, although 41.6% of them considered themselves to be hesitant. More than half of the respondents (54.7%) did not want their children to receive all recommended vaccines. Additionally, 43.4% of respondents had delayed vaccinating their child at least once for reasons other than illness or allergies, and 32% had decided not to vaccinate their child at least once even though the child did not have any allergies or illnesses. Half of the respondents (51.4%) were not sure that following the vaccination schedule was good for their child, and slightly less than half (44.4%) believed that children receive more vaccines than are good for them. Over 60% of respondents were concerned that vaccines are not safe and that their children could suffer serious consequences from them, and they think it would be better if fewer vaccines were administered at the same time. Nevertheless, most respondents trust their child’s doctor and consider it important to be able to discuss vaccination openly with their child’s doctor (Table 2).

### 3.1. CFA for the Original Study

First, we tested the original three-factor solution previously established by Opel et. al. [32]. The factor loadings ranged between 0.150 and 0.807, followed by multiple squared correlations (R2) from 0.085 to 0.842. Item “Q14—It is important that I will be able to openly discuss my concerns about vaccines with my child’s doctor”, belonging to Factor 2—“Safety and efficacy”, revealed a very low loading of 0.15. Other factor loadings were ≥ 0.354. Furthermore, the fit indices indicated mis-specification: χ2 was much too high (1626.7) as well as χ2/df (18.7) while TLI was low (0.890). However, the fit indices RMSEA, SRMR, and CFI were acceptable (0.097, 0.061, and 0.909, respectively).

### 3.2. EFA

To obtain a better fit for the Croatian population, we performed an EFA on our dataset followed by CFA.

**Figure 1:**
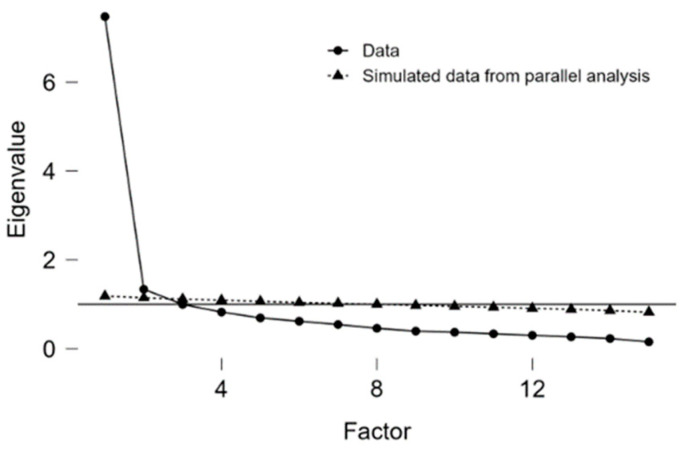
Parallel analysis scree plot of exploratory factor analysis.

The suitability of the training set for factor analysis was supported by the KMO (0.937) and Bartlett’s test of sphericity (*p* < 0.001). A parallel analysis scree plot of EFA is shown in Figure 1. An EFA resulted in a five-dimensional model with factors labeled “Safety” (3 items), “Schedule” (4 items), “Attitudes” (3 items), “Behavior” (2 items), and “Trust” (3 items) (Table 3). Five factors explained 17.6%, 12.8%, 12.7%, 9.6%, and 9.2% of the variance, respectively, resulting in a total of 61.9% of the variance explained. Correlations among factors ranged from 0.569 to 0.752. Factor loadings of the five factors and the uniqueness of the variables are presented in Table 3. Only one item, Q12 (“Overall how hesitant about childhood shots would you consider yourself to be?”), cross-loaded across two factors, “Safety” and “Schedule”, and it was considered to be in the “Schedule” factor where it loaded higher. As shown in Table 3, the highest uniqueness, meaning the smallest relevance of the variable in the factor model, was in question 14: “It is important that I will be able to openly discuss my concerns about vaccines with my child’s doctor”. The majority of fit indices indicated a good model fit (RMSEA = 0.039, SRMR 0.01, TLI = 0.982, CFI = 0.993), while ratio χ2/df indicated an acceptable fit since χ2/df ≤ 3.0 (χ2 = 117.614, *p* < 0.001, df = 40, χ2/df = 2.94).

### 3.3. Confirmatory Factor Analysis for Five-Factor Structure

After an EFA, we performed a CFA to test the five-factor solution on the test data set. The factor loadings ranged between 0.188 and 0.868, followed by multiple squared correlations (R2) from 0.130 to 0.859. Item “Q14—It is important that I will be able to openly discuss my concerns about vaccines with my child’s doctor”, belonging to Factor 5 “Trust”, revealed a very low loading of 0.188, while other factor loadings were greater than 0.381. Furthermore, the fit indices indicated acceptable to good model fit, (χ2/df = 4.3 < 5, TLI = 0.935, SRMR = 0.046, CFI = 0.95, and RMSEA = 0.077).

### 3.4. Discriminant Validity

The HTMT values ranged from 0.650 to 0.888 and were lower than the acceptable level of 0.90, indicating that discriminant validity was established among all constructs.

### 3.5. Reliability

Next, we computed Cronbach’s alpha and McDonald’s omega coefficients to estimate the internal consistency for the whole questionnaire and for each factor separately. Total Cronbach’s alpha was 0.926 (95% CI: 0.920–0.931). Values for each factor were as follows: 0.863 (95% CI: 0.848–0.875) for “Safety”, 0.816 (95% CI: 0.799–0.831) for “Schedule”, 0.805 (95% CI: 0.786–0.822) for “Attitudes”, 0.775 (95% CI: 0.750, 0.799) for “Behavior”, and 0.671 (95% CI: 0.644–0.697) for “Trust”. McDonald’s omega for the whole instrument was 0.931 (95% CI: 0.925–0.936) and the values for particular factors were as follows: 0.871 (95% CI: 0.859–0.883), 0.825 (95% CI: 0.810–0.841), 0.819 (95% 0.802–0.836), 0.775 (95% CI: 0.747–0.805), and 0.746 (95% CI: 0.722–0.771), respectively. Thus, the internal consistency, as measured with Cronbach’s alpha and McDonald’s omega values, ranks as acceptable.

## 4. Discussion

We adapted and translated the PACV questionnaire into Croatian, establishing its validity and reliability as a tool for identifying vaccine hesitancy, primarily among Croatian mothers. Our findings highlight the significance of these results in the context of vaccine hesitancy and the tool’s potential for future use in similar populations. To the best of our knowledge, this is the largest and most comprehensive validation study of the PACV questionnaire performed so far.

The Croatian version of PACV consists of 15 items framed within five distinct factors: “Safety”, “Schedule”, “Attitudes”, “Behavior”, and “Trust”. The obtained five-factor structure for the Croatian population differs from the original research, which identified three factors: “General attitudes”, “Safety and efficacy”, and “Behavior”. However, it should be noted that in the original research, 4 out of 15 items had a relatively strong correlation with two factors, rather than aligning with just one, signaling the need for further investigation into the underlying constructs being measured. This is further supported by the fact that CFA was not conducted in the original study to confirm the obtained factor structure. Originally, the PACV was designed to measure four content domains, including immunization behavior, beliefs about vaccine safety and efficacy, attitudes about vaccine mandates, and trust. So far, several validation studies, with factor analysis included, have been performed worldwide [33,34,35,36,37]. Based on the data provided, it seems that the factor structure of the PACV questionnaire may differ based on geographical and cultural influences. Although a three-factor structure was preferred in most validation studies of PACV, there are several exemptions regarding the number of factors identified and the distribution of items across specific factors. The Malay version [36] identified a four-factor structure, but the authors concluded that a three-factor solution was the most conceptually appropriate and equivalent to the PACV questionnaire. Along with factor domains “General attitudes” and “Safety and efficacy”, a new sub-domain labeled “Schedule and Immunity” was identified. Also, it is worth noting that questions 1 and 2, which form the “Behavior” factor in our results, were deleted in the Malay study. Similar to the Malay study, in the Vietnamese [35] and Arabic [34] studies, four factors were identified; however, the authors concluded that the three-factor solution was the most conceptually suitable. Questions 1 and 2 were deleted as in the Malay study [36], as well as question 4. The Italian version [33] of PACV was quite different from others; it included 11 items under five factors, with items 7 and 15 forming unknown factors. German and French [33] versions of the PACV comprised 14 items and gave factor-loading structures similar to those obtained in the original PACV. Furthermore, the Brazilian study [47] identified only two factors, respectively, called “Behaviors” and “Beliefs”.

To maximize the power of our study, and given the large sample size of this study (*n* = 1814), we divided our data into a training dataset and a testing dataset to conduct both EFA and CFA on the Croatian population. With EFA, we obtained an appropriate factor structure, which we confirmed by CFA. Apart from our study, both EFA and CFA were performed only in the Arabic study [34]. Also, what is important and supports the obtained five-factor structure is that we tested and confirmed discriminant validity, a measure that is used to demonstrate that the obtained factors are independent and unique. Notably, the majority of validation studies have excluded at least one item from the questionnaire due to poor fit. Although no items were excluded in our study during the exploratory step, the CFA indicated poor results for item 14. Item 14 had also poor performance in both the French and Italian versions of the questionnaire [33]. Therefore, future studies should provide analyses of individual items using Item Response Theory, a modern approach that is widely used to assess and improve the measurement properties of questionnaires.

The majority of participants (95.6%) in our study were women. Similarly, in other validation studies of the PACV questionnaire, most respondents were also mothers, ranging from a minimum of 69.2% in the Arabic validation study [34] to a maximum of 92.7% in the Italian validation study [33]. This high percentage of mothers was expected, as women are generally more willing to participate in health-related surveys than men [48], are more involved in child health [49], and in Croatia, mothers tend to take on a greater role in child-rearing and caregiving than fathers [50].

It is concerning that our research identified nearly half of the respondents as vaccine-hesitant parents, although they do not all think of themselves as hesitant. The original study by Opel et al. [32], conducted during the development of the questionnaire in the USA in 2011, found 25% of parents were vaccine-hesitant. The Swiss validation study [33] for the French version of the questionnaire found 40% of parents were vaccine-hesitant, 28% for the German version, and 11% for the Italian version. A meta-analysis published in 2024, which included 23 studies from over 30 countries, found an overall vaccine hesitancy prevalence of 21% [51]. Specifically, for studies using the PACV questionnaire, the prevalence was 27%. Vaccine hesitancy prevalence varied across different countries and studies, ranging from 3.4% to 41.8%. The highest rates recorded with the PACV questionnaire were in Israel (42%), Poland (41%), Ukraine (40%), Lithuania (38%), Italy (36%), Bulgaria (35%), Austria (33%), Greece (33%) [52], and Turkey (31.3%) [53], which still show slightly lower rates than those recorded in our study among the Croatian population. Regarding the number of parents who believe they are hesitant about vaccination, it is slightly lower than the number of parents classified as vaccine-hesitant using the scoring method (41.6% vs. 48.4%). This number is somewhat lower, but still close to the number of parents identified as hesitant in the Franch version validation study, where 44.4% of parents were classified as hesitant [33]. However, in other validation studies, the percentage of parents identified as vaccine-hesitant is lower than in our study (33% in the original Opel et al. study [32], 28.1% in Turkey [37], 27.4% in the German version validation, 13.7% in the Italian version [33], and 15.9% in Vietnam [35]). Unfortunately, increased hesitancy leads to delays and refusals in vaccinating children, reducing population vaccination coverage, which poses a public health risk and undermines herd immunity [1,2].

A large study involving 18 European countries, including Croatia, reported that 62% of surveyed parents in Croatia in 2019 did not consider themselves hesitant about vaccinating their children [52]. Our data indicate a slightly lower percentage of non-hesitant parents, possibly suggesting increased vaccine mistrust and hesitancy among parents in recent years. Additionally, a significant number of parents delay or refuse to vaccinate their children for reasons not related to illness or allergies. More than half of the respondents do not want their child to receive all recommended vaccines, are unsure about vaccine safety, and fear serious side effects. This distrust in vaccine safety is one of the reasons for vaccination hesitancy. Some studies highlight this distrust as a major reason for parental hesitancy [54,55], and a global study conducted in 67 countries identified Europe as the region with the highest levels of vaccine skepticism [1]. Other studies have also noted high levels of concern about vaccine safety and fear of serious side effects [56], and parents who fear vaccines are significantly more likely to choose not to vaccinate their children [57,58]. Moreover, misinformation, misconceptions, and lack of knowledge about vaccination are also reasons for vaccine refusal [59,60,61]. In our sample, a significant number of parents believe that children receive more vaccines than are good for them and that it would be better if they received fewer vaccines at the same time. This high rate of hesitancy can also be linked to the fact that vaccination hesitancy is significantly higher among mothers than fathers [62,63], and the majority of our sample consisted of mothers.

The reasons for these increased rates of vaccine hesitancy in Croatia can vary. In Croatia, childhood vaccination is mandatory and affects school enrollment, but even if parents do vaccinate their children, that does not mean they are supporting the vaccination program. Some studies have shown that parents can still express serious distrust and fear and be hesitant although they vaccinated their children [64,65]. Mandatory vaccination can also lead some parents to refuse vaccination due to a sense of coercion. According to one study conducted in Zagreb, nearly 30% of respondents believe that vaccination should not be mandatory or are uncertain about it [10]. A population study showed that 20% approve of some, but not all, mandatory vaccines, and 11% of parents are willing to refuse mandatory vaccines if they believe it is in the best interest of their children [16]. Previous research has shown that a significant number of parents in Croatia are concerned about vaccine safety, especially when they receive more vaccines at the same time [10]. Additionally, in Croatia, there is a significant presence of misinformation spread through social media, which can lead to concerns about vaccine safety and side effects. Research conducted in Croatia’s capital, Zagreb, revealed that social networks and media influence parents’ attitudes towards vaccination, particularly younger parents [66]. A qualitative study that examined the decision-making process in avoiding mandatory vaccination highlighted that more than half of the respondents believed in or adhered to some conspiracy theories related to childhood vaccination [58]. Research has also shown that mothers who perceive childhood vaccines as problematic are more likely to seek information about vaccines online, particularly on social media [67], which may make them more vulnerable to misinformation circulating on these platforms. Groups of parents who have shown to be particularly vulnerable to the influence of online media on attitudes toward vaccines are women, younger parents, married parents, and those who have more children [68], and our sample consisted mostly of married younger mothers who mostly have two children. Antivaccine posts have also been linked to a rise in vaccine hesitancy among mothers [69]. Another reason could be the increased distrust in public institutions and the government, which set the vaccination regulations and programs. A study examining trust in the healthcare system and doctors in Croatia found that less than 60% of respondents trust the Croatian healthcare system, with the highest level of distrust seen in the most vulnerable groups, who need the most help [70]. Additionally, this research was conducted at a time when we may still be feeling the effects of the COVID-19 pandemic, which led to a decline in childhood vaccination rates [28] and an increase in negative attitudes [17,21] toward vaccination, as well as greater concerns regarding vaccine safety [17,20,21] and vaccine side effects [20], all of which contribute to increased vaccine hesitancy. These factors, coupled with specific parental beliefs—such as skepticism about the number and timing of vaccines—reflect the complex landscape influencing vaccine hesitancy in Croatia.

### Strengths and Limitations

One of the strengths of our research is its large sample size, which increases the reliability and generalizability of the findings. The sample, while convenient, was sufficiently large and included participants from diverse age groups and regions across Croatia. The online survey format allowed access to respondents from all parts of the country, but we acknowledge that certain groups (e.g., those with limited internet access) might be underrepresented, so this sampling method limits the full representativeness of the sample. The second strength is the double back-translation of the questionnaire, which helped to maintain content validity. The third strength is detailed validity and reliability testing. The research rigorously validated the PACV questionnaire using CFA, EFA, and advanced statistical techniques, demonstrating strong reliability and construct validity and confirming the reliability and validity of the Croatian PACV questionnaire.

Limitations include convenient sampling and an online questionnaire, which may lead to selection bias. Also, the online survey distributed via social media may have led to the underrepresentation of some socioeconomic groups with limited access to the internet or people not registered on social media. There could be potential recall bias because parents may forget details about their children’s vaccination history, which may result in inaccurate responses. Also, there is a possibility that participants might provide responses they perceive as socially acceptable, rather than their true attitudes, particularly regarding vaccine hesitancy, which may lead to social desirability bias. Another limitation is gender-related bias, since we have a high proportion of mothers in the sample, and they may be more hesitant than fathers. Additionally, we lack data on the vaccination status of the respondents’ children, preventing us from comparing vaccination rates and hesitancy, such as the percentage of those who are hesitant but still vaccinate their children. Lastly, this study was conducted during a period potentially influenced by the COVID-19 pandemic, which may have introduced additional contextual factors affecting parents’ attitudes toward vaccination.

## 5. Conclusions

This study adapted and translated the original PACV questionnaire into Croatian, on a sample predominantly composed of mothers, establishing it as a valid and reliable tool for identifying vaccine hesitancy. The Croatian version consists of 15 items across five factors: “Safety,” “Schedule,” “Attitudes,” “Behavior,” and “Trust,” differing from the original three-factor structure, which shows the impact of cultural and regional context. This study revealed a high prevalence of vaccine hesitancy among Croatian parents, indicating a public health concern and the need for targeted strategies to improve vaccination rates as vaccine hesitancy not only risks the health of individual children but also threatens community herd immunity. This is precisely why we believe that the newly developed and validated PACV questionnaire for the Croatian population will be a useful tool for healthcare providers, policymakers, and researchers. It can aid in addressing vaccine hesitancy and in developing effective strategies and policies to increase childhood vaccination coverage and rebuild trust in vaccines in Croatia. Future research could conduct a deeper analysis that includes a more representative sample and examine the influence of broader social factors, such as the impact of media, social networks, and misinformation on vaccine hesitancy. Efforts should be made to rebuild trust in healthcare systems and vaccination programs. Policymakers should consider implementing strategies such as programs for vaccinations and monitoring vaccination rates to identify and address disparities promptly. By addressing these areas, researchers and practitioners can work together to develop and implement evidence-based strategies to reduce vaccine hesitancy and improve public health outcomes.

## Figures and Tables

**Table 1:** Socio-demographic characteristics of the participants.

	*N* = 1814
Gender; *n* (%)	
Male	74 (4.1)
Female	1740 (95.6)
Age; mean ± SD (min–max)	35.64 ± 6.32 (21–60)
Marital status; *n* (%)	
Single	16 (0.9)
Married/In a cohabitation relationship	1722 (94.6)
Widow	7 (0.4)
Divorced	696 (3.8)
Educational level; *n* (%)	
Elementary school	2 (0.1)
Highschool	631 (34.8)
Graduate	1035 (57.1)
Postgraduate (Master’s or PhD)	146 (8.0)
Number of children; *n* (%)	
One	640 (35.3)
Two	802 (44.2)
Three	288 (15.9)
Four and more	84 (4.6)

**Table 2:** PACV items in both the original language and their translated versions, along with corresponding descriptive statistics.

PACV Item (Original Language and Translated Croatian Language)	Parent Response ^1^	*N* = 1814
Have you ever delayed having your child receive a shot for reasons other than illness or allergy? [Jeste li ikada odgodili cijepljenje svog djeteta/djece iz razloga koji ne uključuju bolesti ili alergije?]	Yes	809 (43.4%)
	No	1006 (54.0%)
	Don’t know	49 (2.6%)
Have you ever decided not to have your child receive a shot for reasons other than illness or allergy? [Jeste li ikada odlučili ne cijepiti dijete/djecu iz razloga koji ne uključuju bolesti ili alergije?]	Yes	596 (32.0%)
	No	1223 (65.6%)
	Don’t know	45 (2.4%)
How sure are you that following the recommended shot schedule is a good idea for your child? [Koliko ste sigurni da je pridržavanje preporučenog rasporeda cijepljenja dobro za Vaše dijete?]	0–5 ^2^	958 (51.4%)
	6–7	186 (10.0%)
	8–10	720 (38.6%)
Children receive more shots than are good for them. [Djeca primaju više cjepiva nego što je dobro za njih.]	Agree ^3^	828 (44.4%)
	Disagree	515 (27.6%)
	Not sure	521 (28.0%)
I believe that many of the illnesses that shots prevent are severe. [Vjerujem da su mnoge bolesti koje cjepiva sprječavanju ozbiljne.]	Agree	1303 (69.9%)
	Disagree	271 (14.5%)
	Not sure	290 (15.6%)
It is better for my child to develop immunity by becoming sick than to receive a shot. [Bolje je za moje dijete da imunitet stekne prebolijevanjem bolesti nego cijepljenjem.]	Agree	535 (28.7%)
	Disagree	836 (44.8%)
	Not sure	493 (26.4%)
It is better for children to receive fewer vaccines at the same time. [Bolje je za djecu da istovremeno prime manje cjepiva.]	Agree	1147 (61.5%)
	Disagree	281 (15.1%)
	Not sure	436 (23.4)
How concerned are you that your child might have a serious side effect from a shot? [Koliko ste zabrinuti da bi vaše dijete moglo imati ozbiljne nuspojave od primanja cjepiva?]	Concerned ^4^	1178 (63.2%)
	Not concerned	521 (28.0%)
	Not sure	165 (8.9%)
How concerned are you that any one of the childhood shots might not be safe? [Koliko ste zabrinuti da neko od dječjih cjepiva možda nije sigurno?]	Concerned	1123 (60.2%)
	Not concerned	513 (27.5%)
	Not sure	228 (12.2%)
How concerned are you that a shot might not prevent the disease? [Koliko ste zabrinuti da cjepivo možda neće spriječiti bolest?]	Concerned	773 (41.5%)
	Not concerned	736 (39.5%)
	Not sure	355 (19.0%)
If you had another infant today, would you want him/her to receive all the recommended shots? [Ako biste sada dobili novo dijete, biste li željeli da ono primi sva preporučena cjepiva?]	Yes	1020 (54.7%)
	No	549 (29.5%)
	Don’t know	295 (15.8%)
Overall, how hesitant about childhood shots would you consider yourself to be? [Ukupno gledajući, u kojoj mjeri se smatrate nesigurnim/om po pitanju cjepiva za djecu?]	Hesitant ^5^	776 (41.6%)
	Not hesitant	892 (47.9%)
	Not sure	196 (10.5%)
I trust the information I receive about shots. [Vjerujem informacijama o cjepivima koje dobijem od liječnika.]	Agree	902 (48.4%)
	Disagree	546 (29.3%)
	Not sure	416 (22.3%)
I am able to openly discuss my concerns about shots with my child’s doctor. [S djetetovim liječnikom mogu otvoreno raspravljati o mojim brigama oko cjepiva.]	Agree	1640 (88.0%)
	Disagree	110 (5.9%)
	Not sure	114 (6.1%)
All things considered, how much do you trust your child’s doctor? [Uzimajući sve u obzir, koliko povjerenja imate u svog liječnika?]	0–5 ^6^	554 (29.7%)
	6–7	265 (14.2%)
	8–10	1045 (56.1%)

^1^ Responses after sorting into 3 categories: hesitant, non-hesitant, and unsure. ^2^ Response category on a scale of 0–10, with 0 being “not at all sure” and 10 being “completely sure”. ^3^ Agree reflects combined responses of strongly agree and agree; disagree reflects combined responses of strongly disagree and disagree. ^4^ Concerned reflects the combined responses of very and somewhat concerned; not concerned reflects combined responses of not concerned at all and not too concerned. ^5^ Hesitant reflects combined responses of very and somewhat hesitant; not hesitant reflects combined responses of not hesitant at all and not too hesitant. ^6^ Response category on a 0–10 scale, with 0 being “do not trust at all” and 10 being “completely trust”.

**Table 3:** Factor loadings.

	Safety	Schedule	Attitudes	Behavior	Trust	Uniqueness
Q9 How concerned are you that any one of the childhood shots might not be safe?	1.000					0.129
Q8 How concerned are you that your child might have a serious side effect from a shot?	0.949					0.214
Q10 How concerned are you that a shot might not prevent the disease?	0.574					0.473
Q12 Overall how hesitant about childhood shots would you consider yourself to be?	0.405	0.426				0.510
Q7 It is better for children to receive fewer vaccines at the same time		0.664				0.595
Q4 Children receive more shots than are good for them.		0.636				0.256
Q3 How sure are you that following the recommended vaccine schedule is a good idea for your child?		0.445				0.316
Q5 I believe that many of the illnesses that shots prevent are severe.			0.692			0.489
Q6 It is better for my child to develop immunity by becoming sick than to receive a shot.			0.672			0.341
Q11 If you had another infant today, would you want him/her to receive all the recommended shots?			0.475			0.234
Q1 Have you ever delayed having your child receive a vaccine for reasons other than illness or allergy?				0.888		0.303
Q2 Have you ever decided not to have your child receive a vaccine for reasons other than illness or allergy?				0.641		0.356
Q15 All things considered, how much do you trust your child’s doctor?					0.786	0.446
Q14 I am able to openly discuss my concerns about vaccines with my child’s doctor.					0.542	0.826
Q13 I trust the information I receive about shots (from doctors).					0.500	0.228

## Data Availability

The data that support the findings of this study are available from the authors upon reasonable request.
